# The Futalosine Pathway Played an Important Role in Menaquinone Biosynthesis during Early Prokaryote Evolution

**DOI:** 10.1093/gbe/evu007

**Published:** 2014-01-06

**Authors:** Xiao-Yang Zhi, Ji-Cheng Yao, Shu-Kun Tang, Ying Huang, Hong-Wei Li, Wen-Jun Li

**Affiliations:** ^1^Key Laboratory of Microbial Diversity in Southwest China, Ministry of Education and the Laboratory for Conservation and Utilization of Bio-Resources, Yunnan Institute of Microbiology, Yunnan University, Kunming, People’s Republic of China; ^2^State Key Laboratory of Microbial Resources, Institute of Microbiology, Chinese Academy of Sciences, Beijing, People’s Republic of China; ^3^The First Hospital of Qujing City, Qujing Affiliated Hospital of Kunming Medical College, Qujing, People's Republic of China; ^4^Key Laboratory of Biogeography and Bioresource in Arid Land, Xinjiang Institute of Ecology and Geography, Chinese Academy of Science, Ürűmqi, People’s Republic of China

**Keywords:** menaquinone, futalosine pathway, 1,4-dihydroxy-2-naphthoate, horizontal gene transfer, phylogeny

## Abstract

Menaquinone (MK) is an important component of the electron-transfer system in prokaryotes. One of its precursors, 1,4-dihydroxy-2-naphthoate, can be synthesized from chorismate by the classical MK pathway. Interestingly, in some bacteria, chorismate can also be converted to 1,4-dihydroxy-6-naphthoate by four enzymes encoded by *mqnABCD* in an alternative futalosine pathway. In this study, six crucial enzymes belonging to these two independent nonhomologous pathways were identified in the predicted proteomes of prokaryotes representing a broad phylogenetic distribution. Although the classical MK pathway was found in 32.1% of the proteomes, more than twice the proportion containing the futalosine pathway, the latter was found in a broader taxonomic range of organisms (18 of 31 phyla). The prokaryotes equipped with the classical MK pathway were almost all aerobic or facultatively anaerobic, but those with the futalosine pathway were not only aerobic or facultatively anaerobic but also anaerobic. Phylogenies of enzymes of the classical MK pathway indicated that its genes in archaea were probably acquired by an ancient horizontal gene transfer from bacterial donors. Therefore, the organization of the futalosine pathway likely predated that of the classical MK pathway in the evolutionary history of prokaryotes.

## Introduction

In prokaryotes, menaquinone (MK) and ubiquinone (UQ) derived from chorismate are important components of the electron-transfer pathway. These two electron carriers differ in both their biosynthesis (they share only the shikimate pathway) and their redox midpoint potentials (Unden and Bongaerts 1997). The more negative midpoint potential of MKs causes them to be abundant under anaerobic conditions (Meganathan 2001; Sharma et al. 2012). In bacteria, UQs are only found in alpha-, beta-, and gamma-proteobacteria, while MKs occur more widely (Collins and Jones 1981; Søballe and Poole 1999) and also are the only quinone in the early-branching archaeal and bacterial phyla (Schütz et al. 2000). These factors indicate that MKs probably appeared before UQs in the evolutionary history of prokaryotes, when the atmosphere was less oxidizing (before the appearance of oxygenic photosynthesis) and when fewer taxa existed (Schoepp-Cothenet et al. 2009; Nowicka and Kruk 2010). However, some obligatory fermentative bacteria have lost the ability to synthesize MKs, such as most members of the genera *Lactobacillus* and *Streptococcus*, which retain partial genes of the MK biosynthetic pathway (Collins and Jones 1981; Brooijmans et al. 2009).

**Fig. 1.—:**
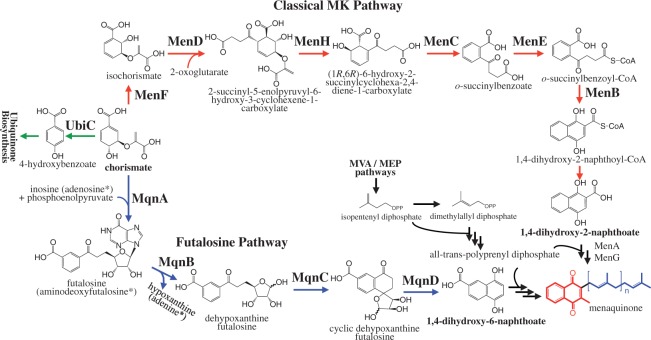
Schematic representation of menaquinone biosynthetic pathways. MenF: isochorismate synthase, including Chorismate_bind (Pfam: PF00425) protein domain; MenD: 2-succinyl-5-enolpyruvyl-6-hydroxy-3-cyclohexene-1-carboxylate synthase, including TPP_enzyme_C (PF02775), TPP_enzyme_M (PF00205), and TPP_enzyme_N (PF02776) domains; MenH: (1*R*,6*R*)-6-hydroxy-2-succinylcyclohexa-2,4-diene-1-carboxylate synthase, including Abhydrolase_1 (PF00561) domain; MenC: *o*-succinylbenzoate synthase, including MR_MLE (PF01188) and MR_MLE_N (PF02746) domains; MenE: *o*-succinylbenzoate-CoA ligase, including AMP-binding (PF00501) domain; MenB: 1,4-dihydroxy-2-naphthoyl-CoA synthase, including ECH (PF00378) domain; MenI: 1,4-dihydroxy-2-naphthoyl-CoA thioesterase, including 4HBT (PF03061) domain; MqnA: futalosine synthase, including VitK2_biosynth (PF02621) domain; MqnB: futalosine hydrolase, including PNP_UDP_1 (PF01048) domain; MqnC: dehypoxanthinyl futalosine cyclase, including Radical_SAM (PF04055) and Radical_SAM_N (PF08497) domains; MqnD: 1,4-dihydroxy-6-naphthoate synthase, including VitK2_biosynth (PF02621) domain. *Adenosine could be a precursor to synthesize aminodeoxyfutalosine via MqnA (Arakawa et al. 2011). The nonpolar moiety of menaquinone is displayed in blue, and the polar moiety is displayed in red.

The biosynthesis of MK involves two precursors: 1,4-dihydroxy-2-naphthoate or 1,4-dihydroxy-6-naphthoate (polar moiety) and an isoprenoid side chain (nonpolar moiety). As shown in figure 1, the polar moiety 1,4-dihydroxy-2-naphthoate is derived from chorismate via the classical MK pathway that recruits six enzymes encoded by *menFDHCEB* genes (Bentley and Meganathan 1982; Meganathan 2001) and 1,4-dihydroxy-2-naphthoyl-CoA thioesterase (Widhalm et al. 2009). Ultimately, 1,4-dihydroxy-2-naphthoate will be converted to MK after the prenylation and methylation catalyzed by polyprenyltransferase (MenA) and methyltransferase (MenG), respectively. However, Hiratsuka et al.(2008) found that some bacteria do not possess *men* homologues and subsequently discovered an alternative pathway, the futalosine pathway, in a nonpathogenic strain of *Streptomyces*. This newly discovered pathway consists of four enzymes encoded by *mqnABCD* genes and some unknown enzymes (Dairi 2009, 2012). In this alternative pathway, chorismate is converted to 1,4-dihydroxy-6-naphthoate through four reactions catalyzed by MqnABCD. Although direct evidence to confirm the synthetic process from 1,4-dihydroxy-6-naphthoate to MK is lacking, the prenylation, methylation, and decarboxylation would be involved in the late step of the futalosine pathway (Hiratsuka et al. 2008).

The primary precursors for the isoprenoid side chain are dimethylallyl diphosphate and isopentenyl diphosphate. There are two distinct synthetic pathways responsible for the synthesis of these precursors: the mevalonate (MVA) and methylerythritol phosphate (MEP) pathways. Recently, Lombard and Moreira (2011) studied the origin and evolution of the MVA and MEP pathways using phylogenomic analyses of a taxon-rich sequence database and concluded that the MVA pathway was likely an ancestral metabolic route in all three domains of life. In other words, if MKs were present in the cenancestor, the MVA pathway led to the biosynthesis of the isoprenoid side chain of MKs. However, which is the ancestral metabolic route for the biosynthesis of the polar moiety of MKs remains unclear. To address this issue, we investigated the distribution of key enzymes in the classical MK pathway and the futalosine pathway in prokaryotes representing a broad phylogenetic distribution. The phylogenetic distribution of genes suggested that the futalosine pathway evolved earlier than the classical MK pathway. These findings have important implications for the nature of MKs in ancestral membranes and their subsequent evolution in prokaryotes.

## Materials and Methods

### Sequence Retrieval

Complete predicted proteome sequences of 1,294 prokaryotes were directly downloaded from the NCBI FTP server (ftp://ftp.ncbi.nih.gov/genomes/Bacteria/ [last accessed January 9, 2014], faa format; see supplementary table S1, Supplementary Material online). To identify each enzyme related to MK biosynthetic pathways, Pfam hidden Markov model (HMM) profiles corresponding to the structural domains of each protein family (see fig. 1 legend) were retrieved from the Pfam database (http://pfam.janelia.org/, last accessed January 9, 2014). Profiling analyses were performed using HMMER v. 3.0 (http://hmmer.janelia.org/, last accessed January 9, 2014). The HMM results were saved in a simple tabular file summarizing the per-target outputs based on the threshold e-value ≤10^−5^. A script was written in Python to manipulate the output from the HMMER analyses, and all protein sequences matching the profile model were collected.

Sequence collections based on protein domains usually contain nontarget sequences that are distantly related to the targets and/or even have distinct functions (e.g., different functional proteins in a superfamily). Thus, we filtered the potential nontargets based on a preliminary phylogenetic analysis of all sequences matching the HMM profile. First, the complete sequence data set was aligned with ClustalW-MPI (Li 2003) and subjected to neighbor-joining tree reconstruction with QuickTree (Howe et al. 2002). Second, all protein sequences were clustered based on their pairwise distances (≤0.3). For each cluster, the sequence with the minimum sum of pairwise distances to other members in the same cluster was selected as a representative. Then, all representative sequences were used as queries to search against the UniRef100 database (http://www.uniprot.org/downloads, last accessed January 9, 2014) using BlastP (Altschul et al. 1990). The functional description of the best hit was used to define the function of all proteins in this cluster. Finally, clusters with unrelated functions were removed, and clusters with target functions were assembled for further analysis. Target sequences with lengths shorter than 100 amino acids were excluded.

### Phylogenetic Analyses

Because MenFBC proteins were identified in more than 480 predicted proteomes, a three-step approach similar to previous work (Canback et al. 2002) was adopted to reduce the number of bacterial sequences for phylogenetic analysis: 1) all protein sequences (full data set) were subjected to maximum likelihood (ML) tree reconstruction with 100 bootstrap replicates in the MPI-parallelized version of RAxML v. 7.3 (Stamatakis 2006), 2) all sequences in the ML tree were clustered based on whether the cluster members showed pairwise distances ≤0.3, were from the same taxonomic lineage (e.g., order), and had sound phylogenetic relationships with each other (≥70% bootstrap support), and 3), all but one representative of each cluster were excluded. All proteins from domain archaea were retained, and all MqnACD sequences were also analyzed. Because these enzymes usually did not have homologues in domain Eukarya, proteins in the same superfamilies but with different functions were used as outgroups for isochorismate synthase (MenF), *o*-succinylbenzoate synthase (MenC), 1,4-dihydroxy-2-naphthoyl-CoA synthase (MenB), and dehypoxanthinyl futalosine cyclase (MqnC). Because futalosine synthase (MqnA) and 1,4-dihydroxy-6-naphthoate synthase (MqnD) belong to the same protein family (Pfam: VitK2_biosynth, PF02621), the phylogeny of the whole protein family was reconstructed.

The reduced data sets were realigned using MAFFT (Katoh et al. 2002), and sites with ambiguous alignments were removed by the Gblocks method in SEAVIEW software (Gouy et al. 2010) with options for a less stringent selection: allow smaller final blocks; allow gap positions within the final blocks; and allow less strict flanking positions. The evolutionary model was selected by a maximum-likelihood approach using ProtTest 3 (Darriba et al. 2011), and the Akaike information criterion. According to the results of ProtTest analysis, LG model (Le and Gascuel 2008) with a proportion of invariable sites and a gamma-shaped distribution of rates across sites (LG+I+G) was selected for MenF and MqnC, and LG+G was selected for MenB, MenC, and MqnAD. The ML trees were reconstructed using RAxML v. 7.3. Nonparametric bootstrap resampling with 1,000 replicates was performed to evaluate the robustness of the tree topologies. Bayesian inference was implemented in BEAST version 1.8.0 (Drummond et al. 2012). The Markov chain Monte Carlo (MCMC) analysis was run until evidence of proper mixing was obtained (up to 5,000,000 generations); the chain was sampled every 500th generation. Data from two independent runs were combined. Results were visualized in Tracer v. 1.5 (http://tree.bio.ed.ac.uk/software/tracer/, last accessed January 9, 2014), and proper mixing of the MCMC was assessed by calculating the effective sampling size (ESS) for each parameter. All ESS values were >100. For each data set, the maximum clade credibility tree, which is the tree with the largest product of posterior clade probabilities, was selected from the posterior tree distribution (after removal of 50% burn-in) using the program TreeAnnotator version 1.8.0 (available as part of the BEAST package).

## Results and Discussion

### Lineage Distributions of MK Biosynthetic Pathways

All 1,294 hypothetical proteomes constructed from their published genomes were interrogated using HMM profiles. Given that this method usually also collected nontarget sequences with different functions, we combined phylogenetic analysis with functional annotation (BlastP against UniRef100 database) to filter target sequences for each enzyme in the classical MK pathway, the futalosine pathway, and the first committed enzyme (chorismate lyase encoded by *ubiC*) for UQ biosynthesis. Even after this step, some enzymes could not be differentiated from their homologs with distinct functions. Finally, in the classical MK pathway, the sequences of MenF, MenC, and MenB were identified and collected successfully. And, in the futalosine pathway, the sequences of MqnA, MqnC, and MqnD were assembled and analyzed.

**Fig. 2.—:**
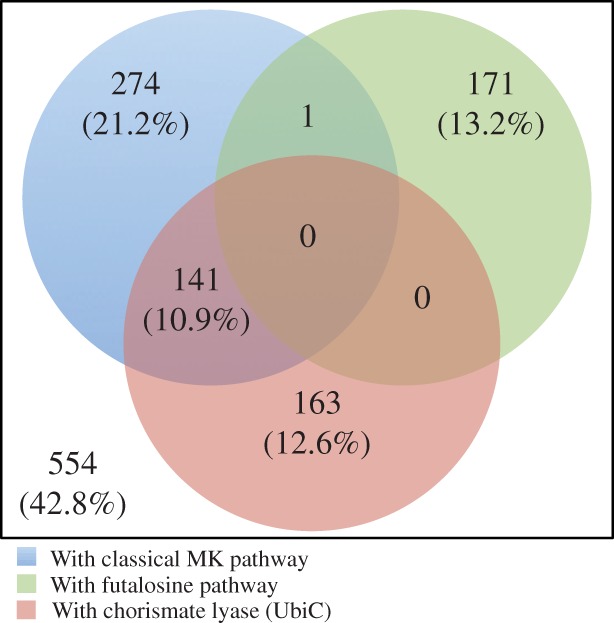
Venn diagram summarizing the distributions of the MQ and UQ biosynthetic pathways and chorismate lyase in 1,294 prokaryotic genomes. In total, 415 (≈32.1%) of the tested prokaryotic genomes contained the classical MK pathway and 172 (≈13.2%) contained the futalosine pathway.

As shown in figure 2, ∼32.1% of the predicted proteomes contained the classical MK pathway, ∼13.2% contained the futalosine pathway, and ∼23.5% contained UbiC. As the first committed enzyme in UQ biosynthesis, UbiC was identified only in alpha-, beta-, and gamma-proteobacteria. Remarkably, only *Stackebrandtia nassauensis* DSM 44728 had both the classical MK pathway and the futalosine pathway. In addition, no bacteria or archaea possessed both the futalosine pathway and UbiC. Although there were 141 predicted proteomes containing both the classical MK pathway and UbiC, nearly all of them were gamma-proteobacteria; the two exceptions were both beta-proteobacteria (candidatus *Accumulibacter phosphatis* and *Dechloromonas aromatica*). These results were consistent with a previous investigation (Dairi 2009).

**Fig. 3.—:**
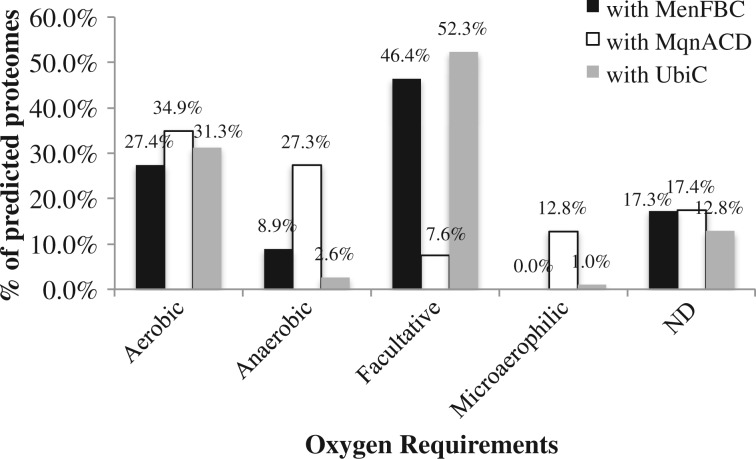
Oxygen requirements of prokaryotes with the MQ and UQ biosynthetic pathways. Most prokaryotes containing enzymes MenFBC of the classical MK pathway were aerobic or facultative. However, 27.3% of the prokaryotes containing enzymes MqnACD of the futalosine pathway were anaerobic. Thus, the majority of MKs in anaerobic prokaryotes was biosynthesized via the futalosine pathway.

Considering that quinones are key elements of the electron transport chain and that the relationship between quinone type and oxygen requirements was thought to be very tight (Maklashina et al. 2006; Bekker et al. 2010), the habitats of all tested prokaryotes were investigated. Their biological properties (oxygen requirement and habitat) were collected from the NCBI Genome Projects page http://www.ncbi.nlm.nih.gov/genome/browse/ (last accessed January 9, 2014). We found that the classical MK and futalosine pathways were correlated with different oxygen requirements (fig. 3). The oxygen requirements of prokaryotes containing the classical MK pathway and UbiC were similar. The majority of them were obligately or facultatively aerobic (73.8%), while few (8.9%) were anaerobic or microaerophilic. In contrast, the oxygen requirements of prokaryotes possessing the futalosine pathway were more diverse, including aerobic (34.9%), anaerobic (27.3%), microaerophilic (12.8%), and facultatively aerobic (7.6%) organisms. Obviously, more prokaryotes living in anaerobic and microanaerobic environments are apt to use the futalosine pathway to synthesize the polar moiety of MKs. Based on the hypothesis that UQ evolved to cope with atmospheric oxygen enrichment, MKs were thought to be an ancient type of quinones. Our results demonstrated that the futalosine pathway contributed substantially to assembling MKs in anaerobes.

We divided the predicted proteomes into 31 divisions based on phylum (table 1). Remarkably, although more predicted proteomes contained the classical MK pathway, they represented a narrower taxonomic range than those containing the futalosine pathway. The futalosine pathway was found in 18 phyla; however, only 11 phyla contained species with the classical MK pathway. Of the five named phyla in the domain archaea, the futalosine pathway could be identified in species from Crenarchaeota, Euryarchaeota, and Thaumarchaeota, but only Euryarchaeota, particularly the family Halobacteriaceae, contained the classical MK pathway. Based on the taxonomic distributions of the enzymes, the most parsimonious explanation is that the last common ancestor of prokaryotes synthesized the polar moiety of MKs using the futalosine pathway, and later, the classical MK pathway evolved in the ancestor of some bacterial phyla. Subsequently, the haloarchaea acquired the classical pathway through horizontal gene transfer (HGT) from bacteria. Also, the putative HGT was also observed in previous studies (Kennedy et al. 2001; Boucher et al. 2003; Baymann et al. 2012; Nelson-Sathi et al. 2012). This hypothesis is consistent with the correlations of the two pathways with oxygen requirements. Generally, identifying the product of HGT rather than vertical descent relies on several lines of evidence, including the distribution of genes in different organisms. In other words, genes that are patchily distributed in several unrelated taxa or in a small subset of a larger phylogenetic group are considered HGT candidates (Ragan 2001). Thus, the classical MK pathway identified in Halobacteriaceae was probably not inherited vertically but acquired through an ancient HGT.

**Table 1:** Distribution of Enzymes Constituting the Classical MK Pathway, the Futalosine Pathway, and UbiC in the Predicted Proteomes of Prokaryotes

Phylum	Number of Proteomes	Quinone Type^a^	Classical MK Pathway			Futalosine Pathway			UK Biosynthesis
			MenF	MenB	MenC	MqnA	MqnC	MqnD	UbiC
Crenarchaeota	30	MK/SQ/CQ/BDTQ			5	10	10	10	
Euryarchaeota	62	—/MK	12	14	15	5	5	5	1
Korarchaeota	1	—			1				
Nanoarchaeota	1	—							
Thaumarchaeota	3	—				1	2	2	
Acidobacteria	5	MK			4	5	5	5	
Actinobacteria	122	MK	89	90	89	15	15	16	
Aquificae	9	—/MTQ				9	9	7	
Bacteroidetes	46	MK	35	35	34	7	4	4	
Chlamydiae	16	MK	2	2	1	14	14	14	
Chlorobi	11	MK/ChQ	11	11	11				
Chloroflexi	15	MK	8	7	8	1	1		
Chrysiogenetes	1	—				1	1	1	
Cyanobacteria	38	MK/PhQ/PQ/α-TQ	37	37	37				
Deferribacteres	3	—				3	3	3	
*Deinococcus-Thermus*	12	MK	1		12	12	12	12	
Dictyoglomi	2	—							
Elusimicrobia	2	ND							
Fibrobacteres	1	—				1	1	1	
Firmicutes	251	MK/DMK	88	94	69	24	25	16	
Fusobacteria	5	—/ND							
Gemmatimonadetes	1	MK	1		1	1	1	1	
Nitrospirae	2	MK			1	1	1	1	
Planctomycetes	5	MK			4	5	5	5	
Proteobacteria	574	MK/DMK/UQ/RQ	210	190	182	66	66	66	303
Spirochaetes	22	—			1	6	6	6	
Synergistetes	2	—							
Tenericutes	35	—							
Thermobaculum	1	—	1	1	1				
Thermotogae	11	—			1				
Verrucomicrobia	4	MK	3	3	2	1		1	

Note.—α-TQ, α-tocopherol quinone; BDTQ, benzodithiophenoquinone; ChQ, chlorobiumquinone; CQ, caldariellaquinone; DMK, demethylmenaquinone; MK, menaquinone; MTQ, menathioquinone; PhQ, phylloquinone; PQ, plastoquinone; RQ, rhodoquinone; SQ, sulfolobusquinone; UQ, ubiquinone; ND, not detected; —, unknown.

^a^The data about the types of quinone collected from references (Collins and Jones 1981; Nowicka and Kruk 2010; Krieg et al. 2011).

Only 57.2% of the proteomes studied represented prokaryotes that could synthesize MK and/or UQ; 42.8% of the predicted proteomes did not have either complete pathway for MK and UQ (fig. 2). In fact, prokaryotes produce other types of isoprenoid quinones (Nowicka and Kruk 2010). Phylloquinone and plastoquinone were discovered in cyanobacteria, isoprenoid quinones with an additional heterocyclic ring containing sulfur occur in Sulfolobales and in thermophilic and aerobic archaea (Lübben 1995), and rhodoquinone is known in purple bacterium (family Rhodospirillaceae). In these organisms with different quinones, only 1 of 38 cyanobacteria genomes, all 11 genomes of the order Sulfolobales, and all 3 genomes of the family Rhodospirillaceae were included in this 42.8% without the complete pathways for MK and UQ. The biosynthesis of phylloquinone is analogous to that of MK (Bouvier et al. 2005), and UQ is a required intermediate for the biosynthesis of RQ in *Rhodospirillum rubrum* (Brajcich et al. 2010). Even if these particular microorganisms are not considered, there still exist many prokaryotes (33.1%) that cannot synthesize any type of isoprenoid quinone. None of the MK pathways were identified in 6 of 26 bacterial phyla (table 1). And among them, Thermotogae, Dictyoglomi, and Synergistetes correspond to anaerobic and mostly nonhost-associated phyla. Moreover, a complete MK pathway only exists in 28 organisms from the 97 archaeal organisms. Even removing Sulfolobales from this analysis, only 42.8% of the archaeal organisms have complete MK pathways, from which 13.1% correspond to halobacterial organisms.

So, there are three potential underlying mechanisms resulting in the nonglobal distribution of quinones in prokaryotes. Fist of all, we found that most of these prokaryotes were anaerobic and host-associated (supplementary fig. S1, Supplementary Material online). The genera *Streptococcus*, *Clostridium*, *Mycoplasma*, *Lactobacillus*, *Staphylococcus*, *Bifidobacterium*, *Rickettsia*, and *Brucella* accounted for 34.6% of the genomes that had lost the quinone biosynthesis pathways. These bacteria live in nutrient-rich environments and obtain their energy from fermentation and/or their host, leading to a progressive loss of respiration genes (Lange et al. 2000; Brooijmans et al. 2009). And then, the extreme paucity of redox driving force also rationalizes the loss of quinone biosynthesis in acetogens and methanogens (other than Methanosarcinales, Schoepp-Cothenet et al. 2013). On the other hand, there are other existing scenarios that quinones only appeared after the divergence of the prokaryotic domains (Lane and Martin 2012; Sousa et al. 2013). According to this assumption, the loss of respiratory gene and host-associated phenomena are not inadequate to explain the absence of MK pathways in phyla that are thought to contain MK (table 1). Nevertheless, the distributions of these two MK biosynthetic pathways basically covered the prokaryotes that use MKs to transfer electrons. Therefore, we hypothesized that the futalosine pathway predated the classical MK pathway, based on the pathways’ taxonomic distributions and correlations with oxygen requirements.

### Phylogenies of the Classical MK Pathway-Related Enzymes

The patchy taxonomic distribution of the classical MK pathway indicated that HGT has played a role in its evolutionary history (table 1). Traditionally, an evolutionary scenario involving HGT was considered to be well supported if the phylogenetic tree for the gene in question disagreed with the accepted organismal tree (Gophna et al. 2006). Although gene duplication followed by gene loss can also result in incongruent tree topologies and systematic phylogenetic artifacts, this criterion is considered the most reliable for establishing HGT (Brown 2003). To examine whether MK pathway in Halobacteriaceae were acquired by HGT, we reconstructed the phylogenies of a reduced set of corresponding enzymes. All sequences from domain archaea were retained to avoid any inaccuracies caused by limited taxon sampling (Pollock et al. 2002; Zwickl and Hillis 2002; Heath et al. 2008).

**Fig. 4.—:**
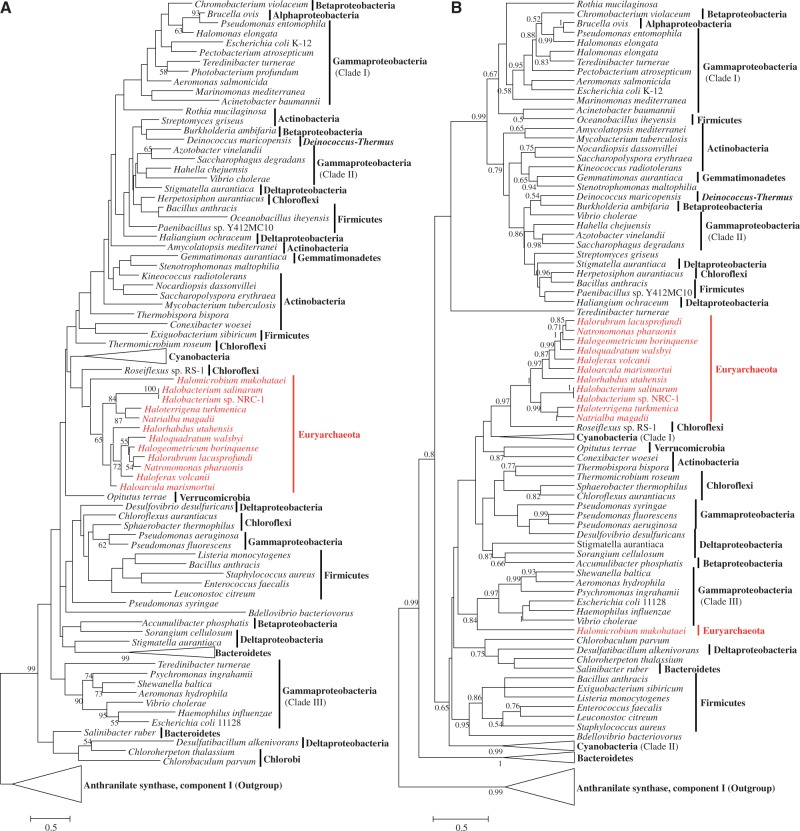
Phylogenies of isochorismate synthase (MenF) based on ML (*A*) and Bayesian (*B*) methods*.* These phylogenies were calculated from an unambiguous amino acid alignment of 146 sequences and 104 sites. Fifty-one sequences of anthranilate synthase (component I), representing 20 phyla, were used as the outgroup. Archaeal species are displayed in red. Bootstrap values above 49% and posterior probabilities above 0.49 are shown as branch labels. The scale bar denotes the number of substitutions per site.

After removing highly divergent and ambiguously aligned blocks, MenF (120 sites) and MenB (218 sites) were analyzed by ML. The phylogenetic trees for MenF are shown in figure 4. The protein sequences from the family Halobacteriaceae formed a distinctive clade (in red) nested within bacteria. Although the bootstrap support was modest (<30%; but in the Bayesian tree, the relationship between haloarchaea and *Roseiflexus* sp. RS-1 was supported by a higher posterior probability [0.95]), probably because of limited phylogenetic signal, its nearest neighbors were the phyla Chloroflexi and Cyanobacteria. This lack of reciprocal monophyly between the two domains is incongruent with the phylogenomic tree of life based on ribosomal proteins (Yutin et al. 2012) and ribosomal RNA (Woese et al. 1990). Remarkably, in the Bayesian tree, *Halomicrobium mukohataei* did not group with other haloarchaea. By comparing the genetic architecture of genes related to the classical MK pathway in Halobacteriaceae (supplementary fig. S2, Supplementary Material online), we found that, unlike other haloarchaea, *Halomicrobium mukohataei* did not have a linked *menF* and *menD*, indicating that *menF* had differing origin in haloarchaea. With this exception, other genes were organized in clusters, which would have facilitated their HGT into the common ancestor of Halobacteriaceae.

**Fig. 5.—:**
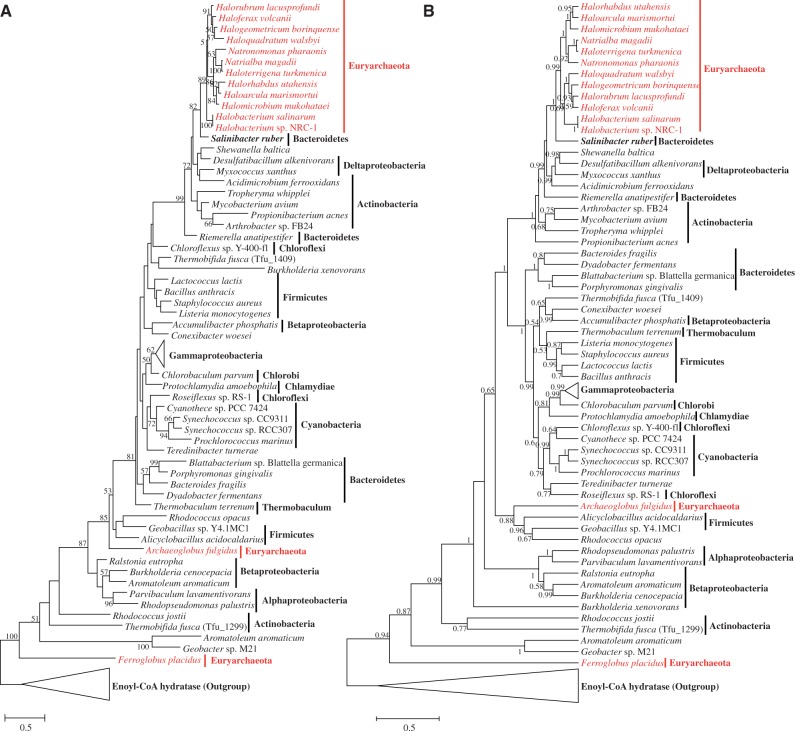
Phylogenies of 1,4-dihydroxy-2-naphthoyl-CoA synthase (MenB) based on ML (*A*) and Bayesian (*B*) methods respectively*.* These phylogenies were calculated from an unambiguous amino acid alignment of 107 sequences and 192 sites. Thirty-three sequences of enoyl-CoA hydratase, representing six phyla, were used as the outgroup. Additional details are described in the legend to figure 4.

As expected, domains bacteria and archaea were very closely related in the phylogenetic trees of MenB (fig. 5). However, in these two trees (based on different methods), Halobacteriaceae formed a well-supported clade with *Salinibacter ruber* (Bacteroidetes), *Shewanella baltica* (Proteobacteria), and some species of the phylum Actinobacteria. Additionally, the MenB from *Archaeoglobus fulgidus* and *Ferroglobus placidus* did not group with other sequences of Halobacteriaceae, implying that MenB had multiple origins in Euryarchaeota. Meanwhile, MenF and MenC were not identified in the predicted proteomes of *A. fulgidus* and *F. placidus* (table 1); this fact suggested that the classical MK pathway was absent in these two species. Although MenC had fewer than 50 homologous sites after removing the highly divergent and ambiguously aligned blocks, we reconstructed its phylogeny based on the full data set (supplementary fig. S3, Supplementary Material online) to compare with those of MenF and MenB. Similar to MenB, the sequences from the family Halobacteriaceae formed a clade with *S. ruber* in the ML tree. Other sequences from phyla Korarchaeota, Euryarchaeota, and Crenarchaeota (except for *Ignicoccus hospitalis*) formed a distinct clade that was also nested within bacteria.

Phylogenies based on rRNA, concatenated proteins, and proteins involved in transcription and translation indicated that the suborders Halobacteriales and Methanosarcinales are sister groups (Brochier-Armanet et al. 2011). Assuming the hypothesis that haloarchaea evolved from methanogens, they had to switch from a strictly anaerobic chemolithoautotrophic lifestyle to an aerobic (photo)organoheterotrophic one (Bapteste et al. 2005). This switch was accompanied by a massive gene gain from the domain bacteria (Kennedy et al. 2001), which may explain why whole-genome-based phylogenetic reconstructions often place them at the base of the archaeal domain (Korbel et al. 2002; Wolf et al. 2002). For example, the haloarchaea are different from other archaea in the oxidization of acetyl-CoA via the methylaspartate cycle (Khomyakova et al. 2011). Instead of using the glyoxylate and citric acid cycles and the ethylmalonyl-CoA pathway, some enzymes that originally belonged to other metabolic pathways from different groups of prokaryotes were recruited by haloarchaea to assemble this patchworked pathway. In addition to substance metabolism, HGT influenced the genetic information processing of haloarchaea as well. One of the two divergent forms of leucyl-tRNA synthetase in haloarchaea was acquired from an organism related to the ancestor of the bacterial domain by an ancient transfer (Andam et al. 2012). Clearly, the haloarchaea were fully equipped with the ability to acquire heterogenous genes by HGT.

Moreover, Rhodes et al. (2010) observed significant HGT between haloarchaea and both halophilic *Salinibacter* bacteria and the thermophilic *Thermotoga* bacteria. In this study, both the MenB and MenC ML trees indicated that haloarchaea were most closely related to *S. ruber*, an extremely halophilic red bacterium found in saltern crystallizer ponds (Anton et al. 2002). They share the same habitat and have similar phenotypes. One mechanism underlying this resemblance was that gene flow occurred between them, although the total number of apparent transfers between *Salinibacter* and haloarchaea appears to be modest (Mongodin et al. 2005). However, the haloarchaea did not group closely with *S*. *ruber* in the MenF tree, indicating that the genes of the classical MK pathway in haloarchaea might have been acquired from different donors. Alternatively, the *menF* gene in *S. ruber* may have undergone complex evolution after the HGT occurred.

### Phylogenies of the Futalosine Pathway-Related Enzymes

**Fig. 6.—:**
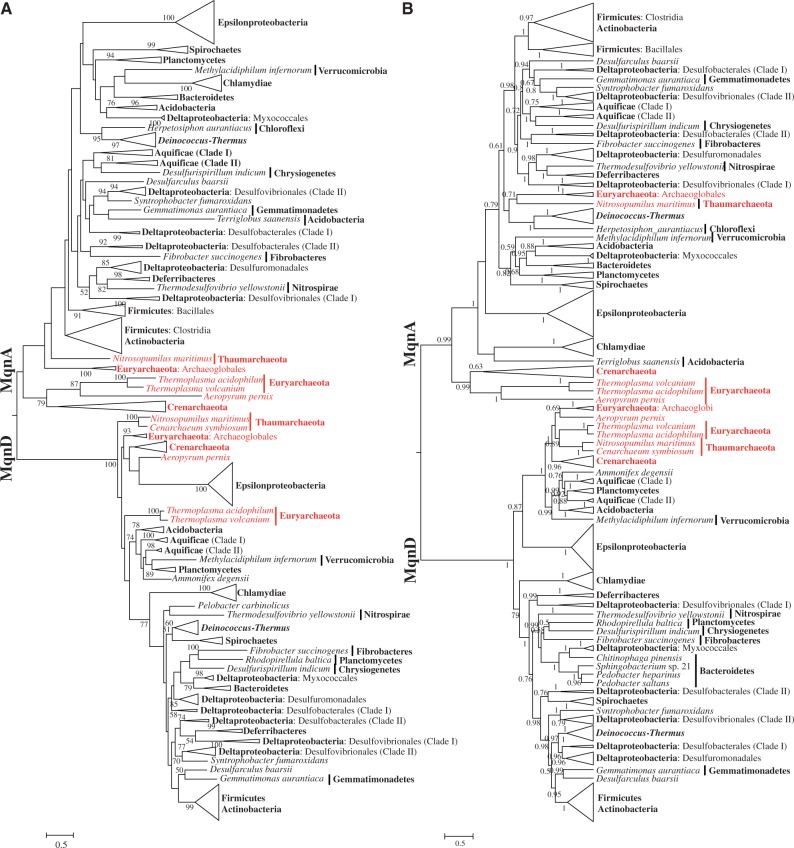
Phylogenies of the protein family (VitK2_biosynth) that includes futalosine synthase (MqnA) and 1,4-dihydroxy-6-raphthoate synthase (MqnD), based on ML (*A*) and Bayesian (*B*) methods. These phylogenies were calculated from an amino acid alignment of 361 sequences and 468 sites (including ambiguous alignments). Additional details are described in the legend to figure 4.

**Fig. 7.—:**
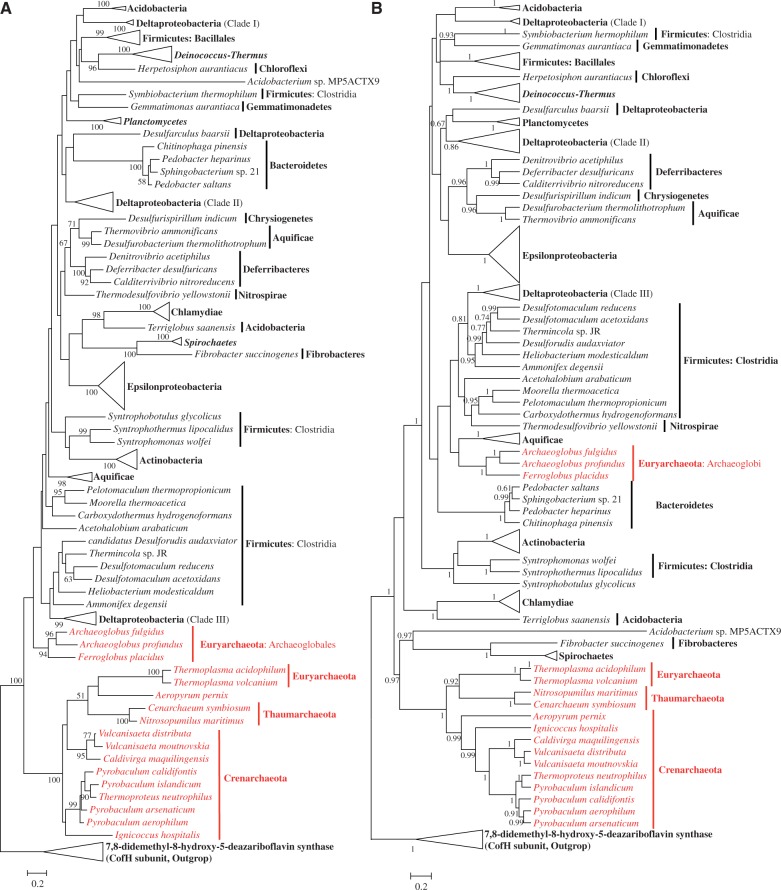
Phylogenies of dehypoxanthinyl futalosine cyclase (MqnC) based on ML (*A*) and Bayesian (*B*) methods. These phylogenies were calculated from an unambiguous amino acid alignment of 200 sequences and 266 sites. Seventeen sequences of 7,8-didemethyl-8-hydroxy-5-deazariboflavin synthase (CofH subunit), representing four phyla, were used as the outgroup. Additional details are described in the legend to figure 4.

The archaeal sequences formed the most ancient branches in the trees of MqnA, MqnD (fig. 6), and MqnC (fig. 7), unlike in the topologies of MenF, MenB, and MenC. The evolutionary relationships between bacteria and archaea in these trees were similar to the phylogenomic tree of life based on ribosomal proteins, although the archaeal groups were not monophyletic. These phylogenetic evidences also support the assumption that the futalosine pathway evolved earlier than the classical MK pathway in prokaryotes. Subsequently, the classical MK pathway was organized in the ancestor of some bacteria. An ancient HGT from bacteria to the common ancestor of the Halobacteriaceae introduced this younger pathway into some archaea.

The futalosine pathway together with the MVA pathway might have been responsible for the biosynthesis of MKs in the ancient prokaryotes. However, 1,4-dihydroxy-2-naphthoate and/or 1,4-dihydroxy-6-naphthoate (DHNA) polyprenyltransferase (MenA), which catalyzes the transfer of a polyprenyl side chain to DHNA, is another essential enzyme that associates the MK biosynthesis pathway with the membrane. Therefore, we investigated the taxonomic distribution of MenA, which belongs to the UbiA protein family (PF01040, data not shown). As expected, most prokaryotes possessed this enzyme, catalyzing the combination of polar and nonpolar moieties of MK, although some taxa lacked it, i.e., phyla Dictyoglomi, Elusimicrobia, Fusobacteria, Spirochaetes, Synergistetes, and Tenericutes and some species *Caldicellulosiruptor*, *Clostridium*, *Lactobacillus*, *Streptococcus*, and *Eubacterium* in phylum Firmicutes. These results were consistent with the distributions of these two MK biosynthetic pathways. In particular, these fermentative and host-associated bacteria have lost genes necessary for respiration. All these data demonstrated that the ancient prokaryotes were fully capable of synthesizing MKs and orienting them toward the cell membrane for respiration.

All these evidences demonstrated that the futalosine pathway was the primordial MK pathway and have appeared early in evolution. However, we still need more evidences to answer the question whether its origin could be dated back to the divergence of domains bacteria and archaea. Although the archaeal sequences occupied the basal positions in the trees of MqnAD and MqnB, they did not form monophyletic clades even within the bacterial domain (fig. 6*B*). The topologies of ML and Bayesian phylogenies differ significantly with respect to positioning of the earliest bacterial and archaeal branches. This suggests that several events of HGT might occur within the evolution of these genes. Additionally, as aforementioned, the quinones might appear after the split of the prokaryotic domains. Thus, these existing evidences merely support that the futalosine pathway predated the classical MK pathway, but it might not originate before the divergence of domains bacteria and archaea.

Compared with Blast and other database search tools, HMM profiling is significantly more accurate for detecting remote homologs. In other cases, however, homologous proteins with different functions might not be differentiated if they have ambiguous phylogenetic relationships. In this study, only some enzymes of these two MKs biosynthetic pathways were identified. Our conclusions are necessarily limited by the methodology. In addition, the high genetic variability of certain proteins, like MenC, led to short sequence lengths after alignment ambiguities were removed, further reducing the utility of the data. However, although the tested proteomes we collected covered all prokaryotic phyla, the uneven taxonomic sampling of genome sequencing projects probably mean that the potential donors in the HGT cannot be confirmed. Additionally, because these enzymes are so ancient and divergent in prokaryotes, long-branch attraction might interfere with the accuracy of the phylogeny. However, none of these limitations negate the hypothesis that the futalosine pathway evolved earlier than the classical MK pathway in prokaryotes, and our findings provide evidence that the cenancestor could biosynthesize MKs.

## Supplementary Material

Supplementary table S1 and figures S1–S3 are available at *Genome Biology and Evolution* online (http://www.gbe.oxfordjournals.org/).

Supplementary Data
