# Supplementary material for: The Futalosine Pathway Played an Important Role in Menaquinone Biosynthesis during Early Prokaryote Evolution
Source: Genome Biol Evol. 2014 Jan 6;6(1):149–60. doi: 10.1093/gbe/evu007 (PMC3914697; doi:10.1093/gbe/evu007)
Supplement: Supplementary Data [file supp_6_1_149__index.html]

The Futalosine Pathway Played an Important Role in Menaquinone Biosynthesis during Early Prokaryote Evolution — Supplementary Data 

# The Futalosine Pathway Played an Important Role in Menaquinone Biosynthesis during Early Prokaryote Evolution

## Supplementary Data

files

**Files in this Data Supplement:**

- Supplementary Data - pdf file
- Supplementary Data - xls file
