# Supplementary figures and images for: The Futalosine Pathway Played an Important Role in Menaquinone Biosynthesis during Early Prokaryote Evolution
Source: Genome Biol Evol. 2014 Jan 6;6(1):149–60. doi: 10.1093/gbe/evu007 (PMC3914697; doi:10.1093/gbe/evu007)

# Supplementary Figure 1

**A**

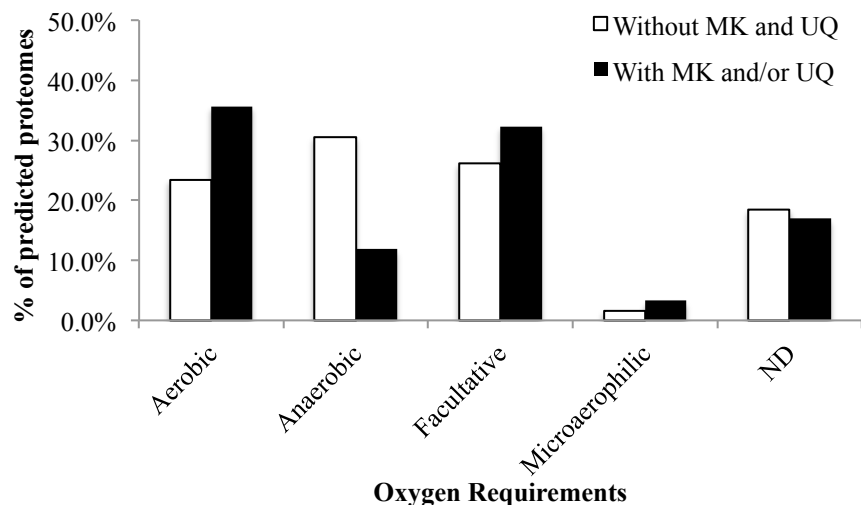

**B**

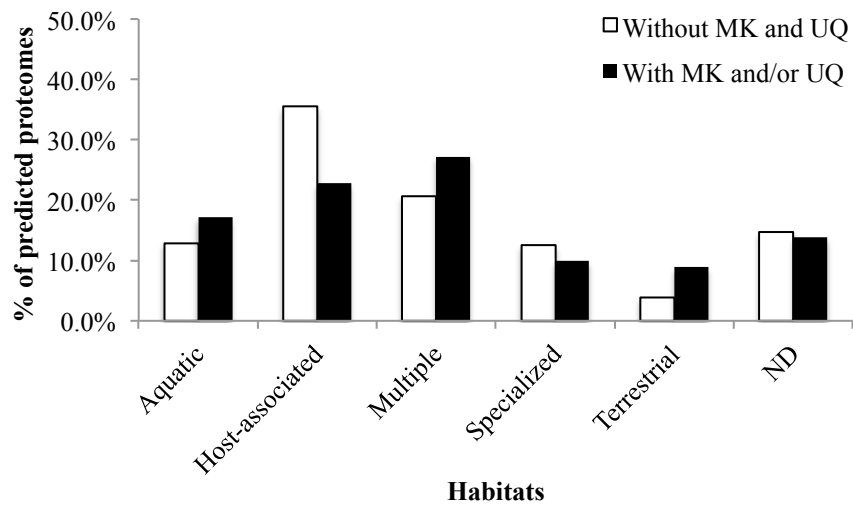

**C**

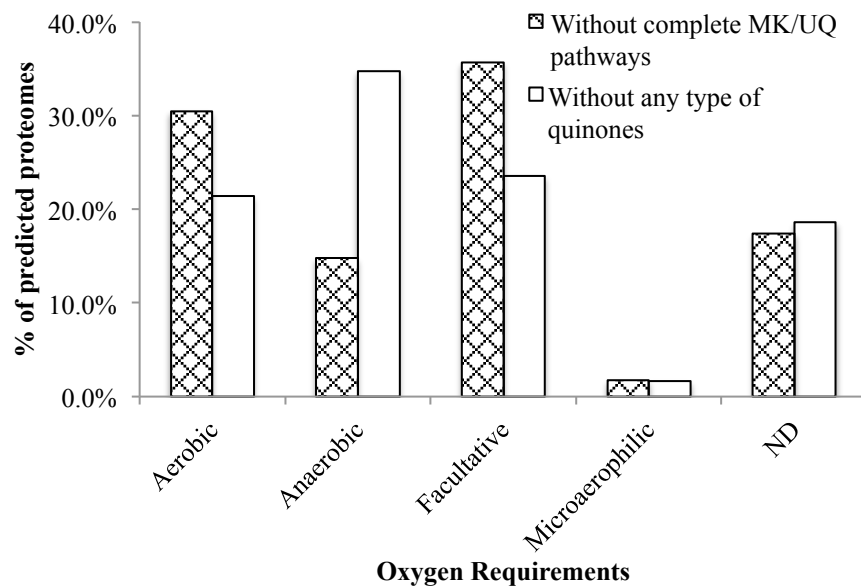

**D**

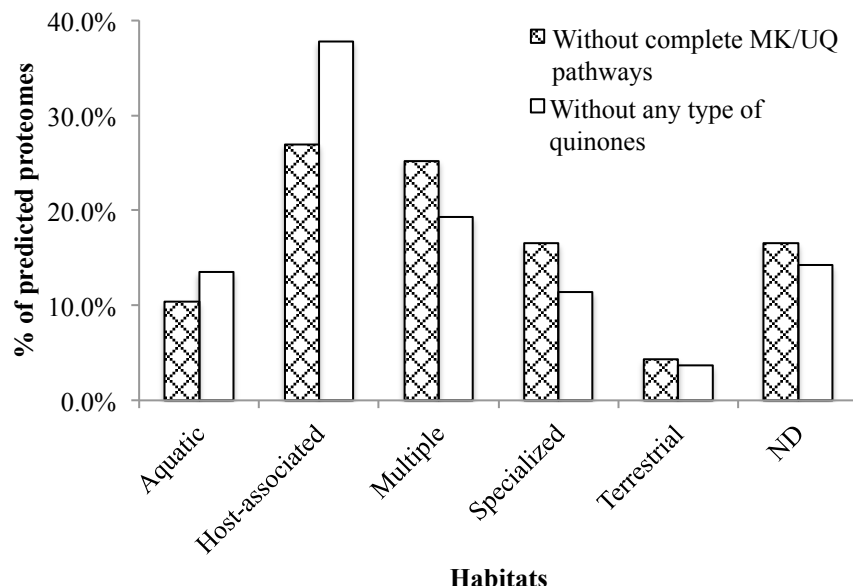

# Supplementary Figure 2

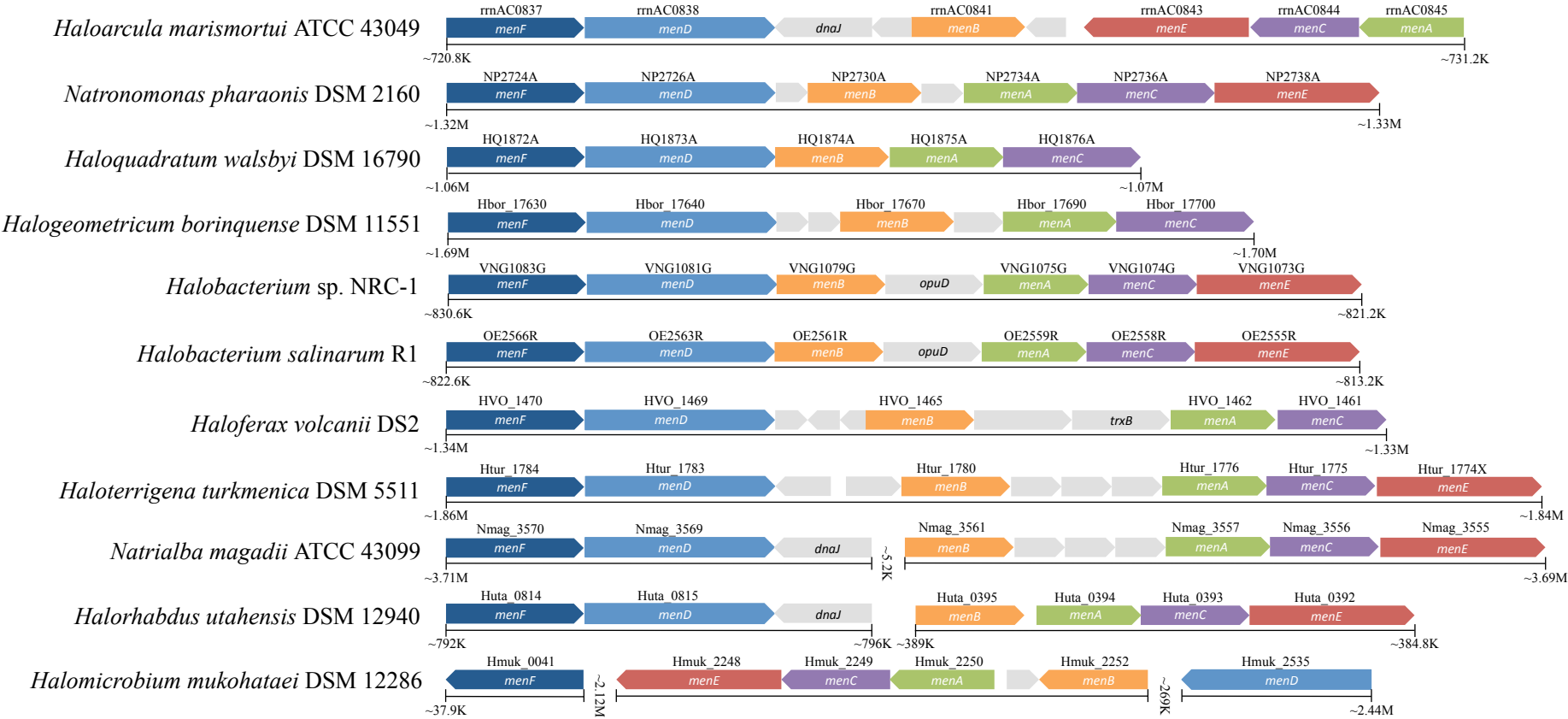

# Supplementary Figure 3

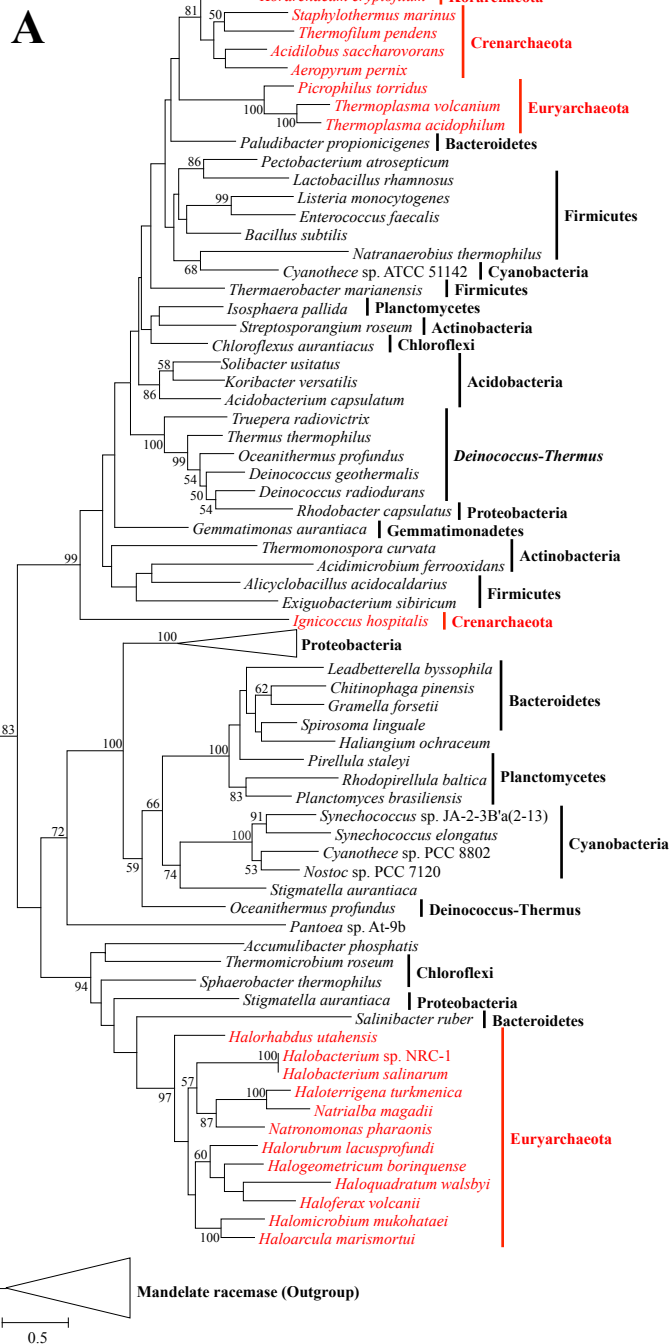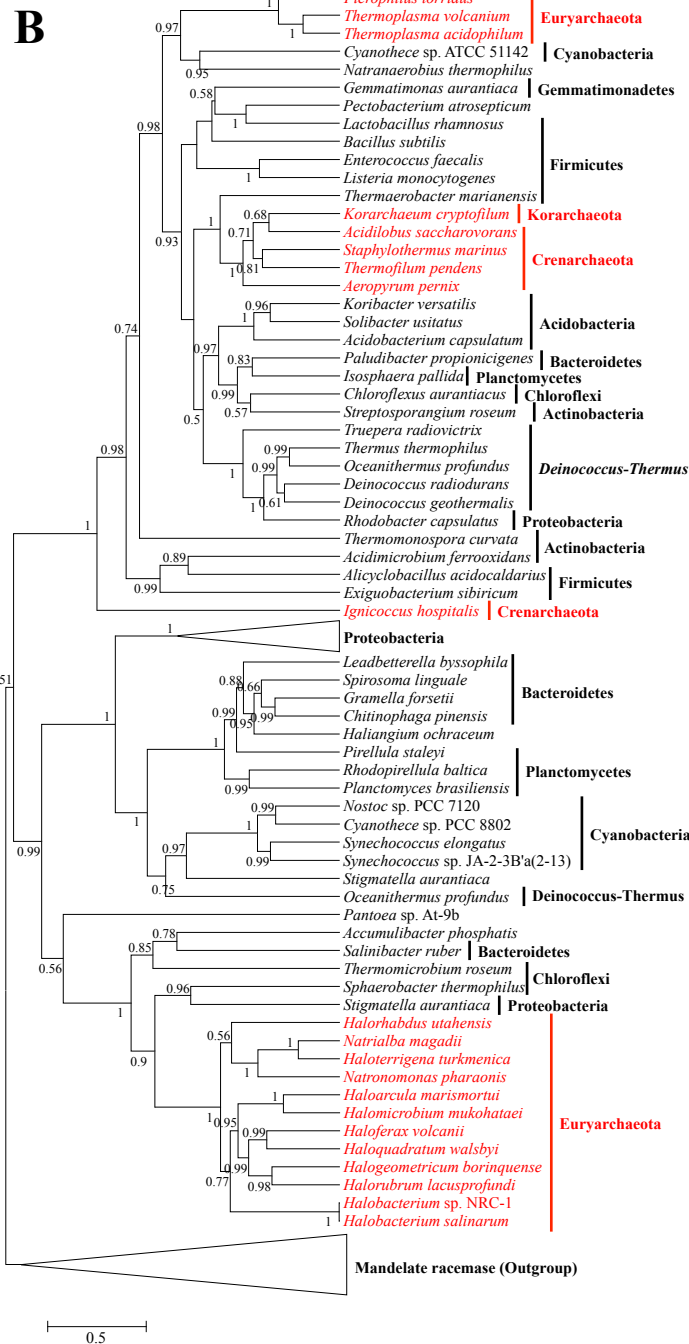

Supplement: Supplementary Data [file supp_evu007_SuppFig-revised.pdf]
